# Mung Bean Starch and Mung Bean Starch Sheet Jelly: NaCl-Based Characteristics Variation

**DOI:** 10.3390/foods12244469

**Published:** 2023-12-13

**Authors:** Shulan Zhou, Tiantian Yuan, Jia Chen, Fayin Ye, Guohua Zhao

**Affiliations:** 1 College of Food Science, Southwest University, Chongqing 400715, China; blueblue@email.swu.edu.cn (S.Z.); yttedu@outlook.com (T.Y.); swaumyh@swu.edu.cn (J.C.); zhaogh@swu.edu.cn (G.Z.); 2 Chongqing Key Laboratory of Speciality Food Co-Built by Sichuan and Chongqing, Chongqing 400715, China

**Keywords:** NaCl, mung bean starch, physicochemical properties, mung bean starch sheet jelly, cooking properties, sensory quality, structural characteristic

## Abstract

Empirical evidence indicates that NaCl can improve the quality of mung bean starch sheet jelly (MBSS) when properly incorporated. In this study, by comparison with a sample without NaCl, the influences of NaCl (1.5–8%, *w*/*w*) on the physicochemical and structural properties of mung bean starch (MBS) and the quality of MBSS were investigated. MBS with added NaCl had greater gelatinization temperature and pasting parameters but lower gelatinization enthalpy than native MBS. With the addition of NaCl, the drying rate of MBSS first accelerated and then declined in the oven-drying process. The addition of NaCl improved the cooking properties of MBSS but decreased the hardness of cooked MBSS. Rheological results implied that the linear viscoelastic region of cooked MBSS decreased with the NaCl addition, and the storage modulus and tan *δ* were more frequency-dependent than the loss modulus of cooked MBSS. The addition of NaCl gradually increased the toughness of dried MBSS and the overall acceptability of cooked MBSS. Furthermore, NaCl decreased the structure order degree of starch in MBSS. Correlation analysis demonstrated that the quality of MBSS had a significant correlation with the molecular and lamellar order of starch. Overall, NaCl could improve the quality of MBSS by regulating the thermal, gelatinizing, and structural properties of MBS.

## 1. Introduction

Edible salts are widely utilized in processed foods, such as meat products, preserved eggs, fruit preserves, baked stuff (including bread, biscuits), noodles, and others [1,2,3]. They acquired the status of a metabolic regulator and were categorized as macrominerals; thus, they are essential to our diet. Edible salts have been used to inhibit microbial growth, promote flavor-forming [4], and ensure the texture, taste, and color in processed meat [2,5,6]. For instance, in bacon processing, salt has facilitated a moderate lipid oxidation by decreasing the activities of antioxidant enzymes such as catalase, glutathione peroxidase, and superoxide dismutase, which has been beneficial to cured flavor development [7]. In breadmaking, the addition of salts improves the strength and stability of dough and the loaf volume of bread [8]. Specifically, the addition of NaCl into noodles has led to an increase in cooking quality but has had only a marginal effect on the springiness of cooked noodles [1]. NaCl has shown multi-technological functionalities for white salted noodles, including improving dough properties, cooking, and eating quality [9]. Meanwhile, alkaline salts (Na_2_CO_3_, K_2_CO_3_), which conferred a bright yellow color to noodle products, were indispensable in the production of yellow alkaline noodles [9]. In practice, salt addition has improved the processing performance of raw material and the quality of the products.

For a variety of starch-containing products, edible salts were commonly used to develop the physical properties and final qualities of these products. Traditionally, liangpi, a starch gel food made from wheat flour or wheat starch, exhibited more chewiness and springiness by adding NaCl. As per the gathered data, NaCl was reported to improve the expansion of the starch during thermal processing, which is beneficial for the formation of the porous internal starch structure of puffed snacks [10]. Norton et al. [11] reported that hot air expansion of potato starch pellets occurred when no salts were added, but the presence of salt did enhance the pellet expansion. Potassium alum was commonly added in starch noodles to maintain structural stability during cooking and improve the quality [12,13,14]. In view of the potential toxicity of aluminum ions, alum is regarded as a processing additive and is used appropriately according to production needs [15,16]. Currently, aluminum should be less than 200 mg/kg in the end product according to GB 2076-2014. Recently, salt was used as a way to modify starch properties [16,17]. Zheng et al. [18] reported that the presence of 150 mmol/L NaCl was favorable for the properties of wheat starch used in 3D printing. Yang et al. [16] found that, when applied to heat moisture-treated potato starch with NaCl as the binding paste, the potato starch noodle had improved cooking qualities and texture.

At present, studies mainly focus on the influence of salts on the properties of starch in order to better control the quality of starch-containing products. It has been proposed that NaCl interacts with starch to form a starch–ion complex, which, in turn, decreases the glass transition temperature of starch, promotes heat transfer, and finally improves starch puffing characteristics [11,19]. Potassium alum has been proven to enhance the texture quality of potato starch noodles and reduce the cooking loss. Li et al. [14] found that potassium alum decreased the leakage of amylose from potato starch by forming aluminum hydroxide colloid to adsorb on the surface of starch granules. Moreover, potassium alum facilitated the hydration of potato starch noodles due to the high ionic strength and water-retaining capacity of Al^3+^ ions [14]. Huang et al. [20] found that a moderate NaCl addition (2–6%) led to the enhanced firmness of waxy rice starch paste, indicating that starch retrogradation was promoted. Kaur et al. [21] reported that the addition of salt decreased the swelling power of water chestnut starch, which led to a reduction in the leakage of amylose. Yan et al. [22] suggested that the setback viscosity of mung bean starch (MBS) was significantly increased by the addition of NaCl (0.06–0.10 mol/L), indicating that the short-term retrogradation of MBS was enhanced.

Empirical evidence indicates that starch noodles benefit from adding salts. Some popular and tasty starch noodles in China have been claimed to contain NaCl. For instance, the content of sodium in Shuangta Longkou vermicelli (Shuangta Food Co., Ltd., Yantai, China) was 20 mg/100 g. The content of sodium in Tongren sweet potato vermicelli (Guizhou Jialijia Agricultural Development Co. Ltd., Guizhou, China) was 97 mg/100 g. Moreover, Xianjia mung bean starch sheet jelly produced by Dingtao Xianjia Starch Product Co. (Dingtao, China) contains 413 mg/100 g of sodium. These applications of salt have great value in the starch noodle industry, but their contribution to processing and the quality of starch noodles has not yet gained enough scientific attention. Therefore, mung bean starch sheet jelly (MBSS) was chosen in this study to investigate the functionality of NaCl related to MBSS quality. By comparing MBS with other commercial starches, we find that MBS is an excellent raw material for making starch noodles, vermicelli, or sheet jelly due to its high amylose content and excellent retrogradation properties [12]. Structurally, MBSS is a type of thin (thickness ca. 1 mm) and dried starch gel. Unlike starch films, MBSS does not contain plasticizers; thus, it is fragile and easy to brake. We hypothesized that NaCl would be positively involved in MBSS making. In this regard, we aimed to unravel the effect of NaCl on the properties of MBS, i.e., the drying characteristics, cooking, and texture quality of MBSS. The result of the present study is expected to provide a reference for the quality-control of starch noodles with respect to adding edible salts.

## 2. Materials and Methods

### 2.1. Materials

Mung bean starch (MBS) (*Vigna radiata* L. Wilczek) was supplied by Qingdao Wanjiaxiang Co. Ltd. (Qingdao, Shandong Province, China). MBS consisted of 86.1 g/100 g carbohydrate, 11.9 g/100 g moisture, and 0.005 g/100 g Na. NaCl was of analytical grade and purchased from Kelong Chemicals Co. Ltd. (Chengdu, Sichuan Province, China).

### 2.2. Physicochemical Characterization of Mung Bean Starch

#### 2.2.1. Thermal Properties

The DSC 4000 (PerkinElmer and Co, Waltham, MA, USA) was used to examine the thermal properties of MBS. The centrifuge tubes (10 mL) were filled with starch–water suspension (30%, *w*/*w*). The sample was allowed to acclimate to ambient temperature overnight in order to fully hydrate the starch. The slurry (~22 mg) was weighed in an aluminum pan and heated at a rate of 10 °C/min from 20 °C to 120 °C [23]. TA Instrument TRIOS version 4.4. 0 was used to analyze the onset (*T*_o_), peak (*T*_p_), conclusion (*T*_c_) temperatures, and enthalpy of gelatinization (Δ*H*).

#### 2.2.2. Pasting Properties

The pasting properties of MBS were measured using a Rapid Viscosity Analyzer (RVA-TecMaster, Perten Instruments, Hägersten, Sweden) according to a previous description [24]. The starch slurry was prepared via dispersion in deionized water (10%, db; 28 g of total weight) and put into a sample test canister. The slurry was equilibrated at 50 °C for 1 min and heated to 95 °C at a rate of 10 °C/min; then, it was kept at 95 °C for 2.5 min and finally cooled back to 50 °C at a rate of 12 °C/min.

### 2.3. Preparation of Dried Mung Bean Starch Sheet Jelly

MBS (90 g) and NaCl (0%, 1.5%, 3%, 5%, and 8% based on starch weight) were dispersed in deionized water (140 g) in order to gain a starch slurry; then, they were transferred to the sealed container. After 24 h of equilibration at room temperature, the starch slurry was poured into a stainless tray. The slurry was evenly distributed after being gently shaken and was then place in contact with the surface of a water bath (85 °C) for 90 s. Thus, the slurry was firmly transformed into a gel-like opaque covering and lost its flowability. After that, the tray was immersed completely in a water bath and kept for 2 min. Later, the tray was removed and then immersed in a room-temperature tap water bath to cool down. Therefore, a translucent MBSS formed on the bottom of the tray [25]. The MBSS was removed from the bottom of the tray, the excessive surface water was wiped up, and the MBSS was dried at 50 °C in the electric thermostatic air-drying oven (DHG-9070, Shanghai Yiheng Technology Instrument Co., Ltd., Shanghai, China) for 4.5 h to obtain the dried MBSS.

### 2.4. Drying Characteristics

In order to describe the drying curve of MBSS, the sample was placed in the electric thermostatic air-drying oven at 50 °C. After 0.5 h, the MBSS was removed from the oven and its weight was immediately measured (*m*_n_, g). This operation was repeated after 1, 1.5, 2, 2.5, 3, 3.5, 4, and 4.5 h. After each experimental weight run, the MBSS were dried at 105 °C for 4 h and weighed (*m*_0_, g). The moisture content of the MBSS was calculated via Equation (1) [26]:

Moisture content (%) = 100 × (*m*_n_ − *m*_0_)/*m*_n._ (1)

### 2.5. Qualities Characteristics of Mung Bean Starch Sheet Jelly

#### 2.5.1. Cooking Properties

The dried MBSS (*m*_1_, g) was cooked in water bath at 95 °C for 8 min. After removing the cooked MBSS, the excess water was carefully wiped off their surfaces by using bibulous paper, and the product was weighed (*m*_2_, g). The cooking water was weighed (*m*_3_, g) after being fully dried in an air-drying oven at 105 °C. The cooking yield and cooking loss of MBSS were calculated using Equations (2) and (3), respectively [27]:

Cooking yield (%) = *m*_2_/*m*_1_ × 100, (2)

Cooking loss (%) = *m*_3_/*m*_1_ × 100. (3)

#### 2.5.2. Textural Properties

To determine the texture of cooked MBSS, the dried MBSS was cooked in a water bath at 95 °C for 8 min. After that, the sample was placed parallel on a flat mental plate for the test. The TA.XT plus texturizer fitted with a cylindrical probe (diameter 36 mm, P/36) was run in TPA mode. The instrument settings were as follows: trigger force of 5.0 g; compression ratio of 70%; the speed before test, test speed, and speed after test were 0.8 mm/s, 0.8 mm/s, 0.8 mm/s, respectively; the two compression intervals were 1 s; and each sample was compressed twice continuously and measured at least three times [28].

#### 2.5.3. Rheological Properties

Using a rheometer (DRH-2, TA Instruments, New Castle, DE, USA) fitted with parallel plates (diameter 40 mm, 0.5 mm gap), the rheological characteristics of MBSS were assessed. The samples made in Section 2.3 were moved to the parallel plate for the test after cooking. The linear viscoelastic region (LVE) of the sample was obtained via strain sweep experiments (strain range: 0.01–100%) at 25 °C, 1 Hz. Preliminary strain sweep studies validated the dynamic oscillatory rheological properties of MBSS, which were verified at a frequency of 0.1 to 25 Hz at a strain of 1% (within LVE). The value of energy storage modulus (*G*′), loss modulus (*G*′′), and loss angle tangent (tan *δ*) were obtained [29]. 

#### 2.5.4. Sensory Analysis

Quantitative descriptive analysis (QDA) was conducted to assess sensory characteristics on a scale of 0 to 10 points [30]. The scoring standard was referred to as T/AHFIA051-2020 Tongguan Sheet Jelly and slightly adjusted. Prior to the analysis, every panelist acknowledged informed consent, and their privacy and rights were respected. The sensory panel consisted of twenty assessors (ten male and ten females, aged 21~27) who were trained. Both dried and cooked MBSS were the subjects of the sensory investigations. MBSS samples after drying were directly evaluated for their toughness by breaking by hand. For MBSS after cooking, the samples prepared in Section 2.3 were cooked in a water bath at 95 °C for 8 min; then, they were picked up and put in five disposable odorless plastic bowls by random digits to be assessed for sensory parameters. The sensory test questionnaire was tested using a descriptive test based on certain standards and scores indicated in Table A1 in order to prevent individual differences among the assessors [31].

### 2.6. Determination the Starch Structure of Mung Bean Starch Sheet Jelly

#### 2.6.1. Attenuated Total Reflection—Fourier Transform Infrared Spectroscopy (ATR—FTIR)

ATR-FTIR spectra of MBSS were obtained by a Spectrum Two with a universal ATR sampling accessory. With air as the backdrop, 4 scans were performed at a resolution of 4 cm^−1^, ranging from 400 cm^−1^ to 4000 cm^−1^ [16]. OMNIC 8.0 software (Thermo Nicolet Corp., Erie, PA, USA) was used to deconvolute the obtained data.

#### 2.6.2. X-ray Diffraction (XRD)

The dried MBSS samples were ground to pass a 200 mesh sieve and placed in a dryer to balance moisture for 24 h. The X’Pert3 Powder XRD equipment (PANalytical, Almelo, The Netherlands) was used to examine the starch crystalline structure of MBSS. For the X-ray diffractometer, the Cu-Kα radiation power was 1600 W (40 kV × 40 mA). At a scanning rate of 2°/min, each sample was scanned at a diffraction angle (2*θ*) ranging from 4° to 40°. The relative crystallinity was calculated as the ratio of the crystallinity area to the overall diffraction area by MDI-Jade 6.0 software [32].

#### 2.6.3. Small Angle X-ray Scattering (SAXS)

The SAXS experiment was conducted at 1W2A SAXS station, which is located at the Beijing Synchrotron Radiation Facility (BSRF). Prior to analysis, the dried MBSS samples were ground to pass a 200 mesh sieve. Then, the starch was suspended in deionized water to produce starch slurry (40% starch concentration) [33]. The starch slurry was put into a square slice with a 5 mm diameter round hole in the middle, and both sides were sealed with transparent tape to attain sample thickness of about 1 mm. The sample was placed on the SAXS test platform for testing. The test conditions were as follows: room temperature; the Mar165 CCD detector was used; the active area was 165 mm; the pixel size was 79 μm; the electron energy was 2.5 GeV; the beam current was about 180 mA; the distance between the sample and the detector was 1718 mm; for the incident light, λ = 1.54 nm; scattering vector, *q* (nm^−1^), was defined as *q* = 4πsin(*θ*)/λ, where 2*θ* was the scattering angle, *q* = 0.1–2.5 nm^−1^; and the scattering intensity was recorded via photodiode. In the actual measurement process, two scattering curves were obtained, one was the sample scattering curve, and the other was the background scattering curve. The data were converted into a 2D image by Fit2D (v10.132) software; then, they were background subtracted and analyzed by Origin Pro 2021 (OriginLab Corporation, Northampton, MA, USA).

### 2.7. Statistical Analysis

The tests were carried out in triplicate and the results were reported as means ± standard deviation. The profiles and correlation analysis were using Origin Pro 2021. One-way analysis of variance (ANOVA) with Duncan’s multiple-range tests was used to check the differences (significantly different at *p* < 0.05) through SPASS 25.0.

## 3. Results and Discussion

### 3.1. Effect of NaCl on the Properties of Mung Bean Starch

#### 3.1.1. Thermal Properties

**Figure 1:**
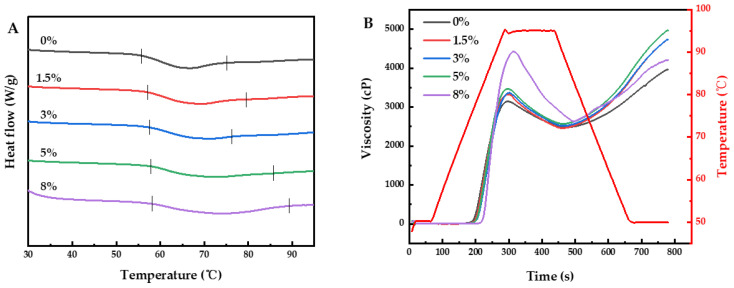
DSC thermograms (**A**) and RVA profiles (**B**) of MBS with different NaCl additions.

DSC is a common tool with which to study the thermal properties of starch, and the results are shown in Figure 1A and Table 1. Depending on the DSC curves, the native MBS showed a typical endothermic peak around 66 °C. By increasing the NaCl content, the *T*_o_, *T*_p_, and *T*_c_ of MBS all increased gradually. This was possibly due to salt ions having a strongly electrostatic interaction with water molecules, which decreased the free water availability for starch gelatinization [34]. Day et al. [35] reported that increasing the level of NaCl increased the gelatinization temperatures as observed via DSC. Similar increases in gelatinization temperature were reported for potato starch [16], tapioca starch [36], and proso millet starch [37]. These studies concluded that the increase in gelatinization temperature was induced by lowering the water activity due to the addition of NaCl [37]. In addition, the gelatinization temperature range (Δ*T = T*_c_ *– T*_o_) was increased from 19.19 °C to 31.06 °C after the addition of 8% (*w*/*w*) NaCl. Typically, an enhanced Δ*T* indicated an increase in structural heterogeneity of starch double helices or entanglements [34]. As closely examined, *T*_c_ increased more than *T*_o_ when the NaCl level increased from 0% (*w*/*w*) to 5% (*w*/*w*). This situation was associated with less-stable double helices melting at the lowest temperature (i.e., *T*_o_) and more residual double helices/entanglements with better quality melting at higher temperatures (i.e., *T*_c_) [34]. Li et al. [34] found that the Δ*T* of wheat starch increased in the presence salts and suggested that the structural heterogeneity of the starch was increased by salts. In terms of the Δ*H*, the effect of NaCl was insignificant when its concentration ranged from 1.5% to 5% (*w*/*w*). However, gelatinization enthalpy (Δ*H*) was significantly lowered as the concentration was up to 8% (*w*/*w*). Normally, Δ*H* reflected the amount of imperfect crystallinity and the ordered structures in starch [16]. As reported by Yang et al. [16], a reduction of Δ*H* was observed in potato starch with the addition of sodium salts (10%, *w*/*w*). Chen et al. [23] found that the Δ*H* values of both lotus seed starch and corn starch increased with the NaCl addition from 2.5% to 10% (*w*/*w*) and decreased with the NaCl addition from 10% to 20% (*w*/*w*). The results indicated that the change of Δ*H* was NaCl-concentration-dependent in some cases. It is known that sodium ions are considered to be structure stabilizers, tending to protect the hydrogen bonds between starch–water molecules and starch–starch molecules to some degree, causing an increase in Δ*H*, while chloride ions are structure destabilizers due to their larger diameter and greater polarization, causing a decrease in Δ*H* [37,38]. At low concentrations (0–5%, *w*/*w*), the stabilizing effect of sodium ions was equivalent to the destabilizing effect of chloride ions, so the effect of NaCl on Δ*H* was insignificant. When the concentration increased to 8% (*w*/*w*), the starch–water system was mostly affected by the more polarized ions, so the NaCl solute had an overall structure-breaking effect, thus decreasing Δ*H*.

#### 3.1.2. Pasting Properties

As shown in Figure 1B, the pasting curve of MBS in excess water was altered with the addition of NaCl. It showed that as NaCl concentration increased from 0% to 8% (*w*/*w*), the time taken to reach starch peak viscosity (TTPV) increased from 4.97 to 5.24 min. It is likely that the addition of NaCl inhibited, to some extent, the initial water from entering the starch granules via electrostatic screening, thus enhancing the resistance potential against swelling and extending TTPV. In addition, NaCl (3%, 5%; *w*/*w*) could significantly increase the pasting temperature (PT) of MBS (*p* < 0.05), while lower (1.5%, *w*/*w*) or higher (8%, *w*/*w*) levels of NaCl had a marginal effect on the PT. A similar trend was reported in potato starch–water mixture [39], in which NaCl inhibited starch gelatinization and increased PT in its low concentration (0–0.5 mol/L) but promoted starch gelatinization and reduced PT in its high concentration (0.5–4 mol/L). Moreover, Luo et al. [40] found that the PT of corn starch with 0.05M NaCl was higher than the native corn starch, but it tended to decrease with the high concentration of NaCl, suggesting that the chloride ions promoted gelatinization by affecting the formation of hydrogen bonds in starch gel [40]. Alteration of PT indicated a change in resistance potential against swelling. Even a small contribution in starch granule swelling could result in an increase in the viscosity of the starch–water mixture [35]. Chloride anions were considered gelatinization factors [41]. At a high level of NaCl (8%, *w*/*w*), the impact of chloride ions on PT was more dramatic than that of sodium ions. Furthermore, as NaCl concentration increased, a continued increase in the peak viscosity (PV, from 3162.5 cP to 4428.7 cP) was observed. Zheng et al. [18] reported that the PV value of wheat starch increased as the levels of NaCl increased from 50 mM to 200 mM. Chen et al. [23] found that a continuous increase in PV occurred in lotus seed starch and corn starch as the NaCl levels increased from 2.5% to 20% (*w*/*w*). Several factors affected PV, such as water uptake, granules swelling, and the interaction between swollen granules, non-gelatinized granules, and leaching amylose. In the present study, the increase in PV indicated an increase in the swelling capability of starch granules, which may be due to the entry of hydrated sodium ions into the starch granules. This not only enhanced the water holding capacity but also strengthened the hydrogen bonding among starch molecules, thereby exacerbating the swelling degree of starch granules. Moreover, salt ions in the continuous phase might interact with leaching amylose, thus reducing the fluidity of starch paste. The trough viscosity (TV) was not significantly affected by NaCl. However, increasing NaCl concentrations led to an increase in breakdown (BD), suggesting that with continued stirring, the granular disintegration of MBS was promoted by adding NaCl. The final viscosity (FV) represented the viscosity of starch paste upon cooling. The setback (SB) reflected that the viscosity of starch paste increases during cooling [40]. As shown in Table 1, salt ions significantly affect FV and SB. The FV and SB of MBS with 1.5% NaCl was 19.23% and 15.25% higher than the FV of MBS without NaCl, respectively. Further increasing the NaCl concentration resulted in a significant decrease in FV (4206.0 cP) and SB (1572.7 cP), but this was still significantly higher than the sample with 0% NaCl (*p* < 0.05). Probably, NaCl reduced the fluidity of starch paste by reducing the intermolecular repulsion; thus, the formation of starch gel networks was promoted [40]. However, a high level of NaCl (8%, *w*/*w*), to some extent, exhibited a salt-out effect by reducing water activity, in which the hydration of starch molecules was inhibited in the hot paste state. Consequently, the formation of starch gel networks was hampered upon the cooling of the paste. The results indicated the concentration-dependence of NaCl on the pasting characteristics of MBS. 

### 3.2. Drying Characteristics and Properties of Mung Bean Starch Sheet Jelly

**Figure 2:**
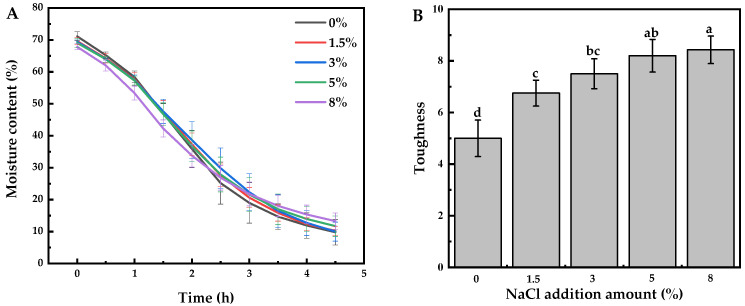
(**A**) Drying curves of MBSS with different NaCl additions. (**B**) The effect of NaCl addition amounts (0–8%) on the toughness of dried MBSS. (Diffenrent lowercase letters (a–d) above the bar in the (**B**) indicate significant differences among the samples at a 0.05 level of confidence.).

Figure 2A depicts the change of moisture content in MBSS during drying with different NaCl additions. As seen from the curves, the moisture content of the samples showed a decreasing trend with extending drying time. The slope of the curve indicates a rapid drying rate of MBSS in the first drying period (0–2.5 h), which tended to gradually slowdown in the latter stages. During the first stage, the moisture content decreased rapidly from 71.13% to 25.16% (*w*/*w*). Furthermore, the water migration was accelerated with the addition of NaCl at this stage. This indicated that the osmotic pressure-driven water migration favored increasing NaCl concentrations [42]. Moreover, the salt ions might bind strongly with water molecules that could be of benefit to the reduction of the driving force of dehydration. During the hindered drying phase, the drying rate gradually decreased until it reached a constant after 4.5 h. It was noted that the addition of NaCl cause the drying rate to decline in this drying period. It was observed that MBSS with 8% (*w*/*w*) NaCl took longer to reach the equilibrium condition than MBSS with less or without NaCl. This was probably due to the reduction of water activity by NaCl. Also, NaCl induced the formation of a dense outer layer of MBSS, which might lead to a decrease in the moisture evaporation rate.

Mechanical properties are essential for dried MBSS. When the dried MBSS was too brittle, it would be broken into small pieces upon handling. Consequently, it could lead to product yield reduction and an increase in packaging requirements and logistics costs. The toughness of dried MBSS was expressed as the resistance to fracture when subjected to a force that causes it to deform. As shown in Figure 2B, the dried MBSS without NaCl had poor toughness and was prone to breakage. However, the toughness of dried MBSS was significantly increased by the addition of NaCl; the dried MBSS could be almost considered a dried starch gel. The dried starch gel would be very brittle due to massive inter- and intra-molecular interactions [43]. To some extent, NaCl prevented the recrystallization of starch and reduced its crystallinity, thus improving its deformation resistance and making it hard to break. Meanwhile, moisture in the dried starch gel could act as a plasticizer, which made the stress successfully distribute throughout system, thus successfully enhancing the sample’s toughness [44]. The moisture in dried MBSS gradually increased as the addition of NaCl increased (Figure 2A), which contributed to improving the toughness of dried MBSS.

### 3.3. Cooking and Texture Qualities of Mung Bean Starch Sheet Jelly

Cooking quality is the important quality characteristic of dried MBSS, and cooking yield and cooking loss represent serious bottlenecks restricting the development of the dried starchy product in industry at present. The cooking yield of MBSS steadily increased as the NaCl concentration increased (Table 2). The cooking yield of MBSS with 8% (*w*/*w*) NaCl was 78.7% greater than that of MBSS without NaCl. In the process of cooking, the MBSS absorbed water and swelled, and NaCl improved the water holding capacity by attracting hydrated ions electrostatically bound inside starch gel networks. Moreover, the dissolution of NaCl created a high-osmotic-pressure condition inside the gel network, which promoted water penetration and absorption into the inner part the gel and enhanced the cooking yield. As listed in Table 2, the cooking loss of MBSS without NaCl was 0.23%. With the addition of NaCl, the cooking loss increased by several times. The main reason for this was the dissolving of NaCl and the leaching of starch molecules. In comparison with the samples containing 1.5% and 3% (*w*/*w*) NaCl, their cooking loss was comparable (*p* > 0.05) and 8.5 times greater than the cooking loss of MBSS without NaCl. NaCl is almost distributed in the gel networks and bound to the amorphous region of MBSS. It could be inferred that NaCl in lower concentration, exhibiting a salt-in effect, disrupted the intermolecular hydrogen bonding among starch molecules, thus promoting the leaching. As the NaCl concentration increased, an increase in the turbidity of the cooking soup was observed. However, according to the study of Yang et al. [16], in the presence of NaCl (10%, *w*/*w*), the cooking loss of potato starch noodles was significantly reduced from 8.56% to 6.15%. They explained that NaCl could inhibit the re-crystallization behavior of amylopectin and the molecular order of starch gel and concluded that the rigidity of retrograded starch gel was improved, thus lowering the cooking loss. However, the case of potato starch noodles with less NaCl addition was not investigated in their work. 

Table 2 summarizes the texture property parameters of MBSS with different NaCl addition levels. Hardness and springiness are the necessary characteristics of starch-gel-based foods. The amount of NaCl clearly influenced the hardness of cooked MBSS. The hardness of samples with 0%, 1.5%, and 3% NaCl showed no significant (*p* > 0.05) differences, while the hardness of samples with 5% NaCl and 8% NaCl was 13.8% and 24.9% less than that of samples without NaCl, respectively. According to the results reported by Yang et al. [16], the hardness of potato starch noodles was significantly enhanced with NaCl addition. They explained that NaCl lowered the swelling power of potato starch and amylose leaching, and that NaCl facilitated the formation of a rigid gel network in the noodle. Huang et al. [20] found that the firmness of waxy rice paste was evidently increased by NaCl. Zhuang et al. [17] found that NaCl increased the potato starch gel strength in relatively low addition levels (0.005 mmol/g), while NaCl showed no significant influence in high addition levels (0.01, 0.02, and 0.04 mmol/g). They explained that the hydrogen bonds in potato starch gel could be enhanced in low-cation conditions but destroyed in high-cation conditions. In our work, NaCl significantly enhanced the water-holding capacity of the cooked MBSS, thus, to some extent, softening the gel networks of the cooked MBSS. Springiness indicates the ability of gel food to return to its original shape after compression. In the present study, the addition of NaCl showed no significant (*p* > 0.05) impact on the springiness of cooked MBSS. Zheng et al. [18] reported that the presence of NaCl showed a marginal (slightly increased or unchanged) effect on the springiness of the wheat starch gels. However, these results were different from the results of Yang et al. [16], who reported that NaCl increased the springiness of potato starch noodles. The different trends might be ascribed to differences in starch sources and end products processes.

### 3.4. Rheological Properties of Cooked Mung Bean Starch Sheet Jelly

**Figure 3:**
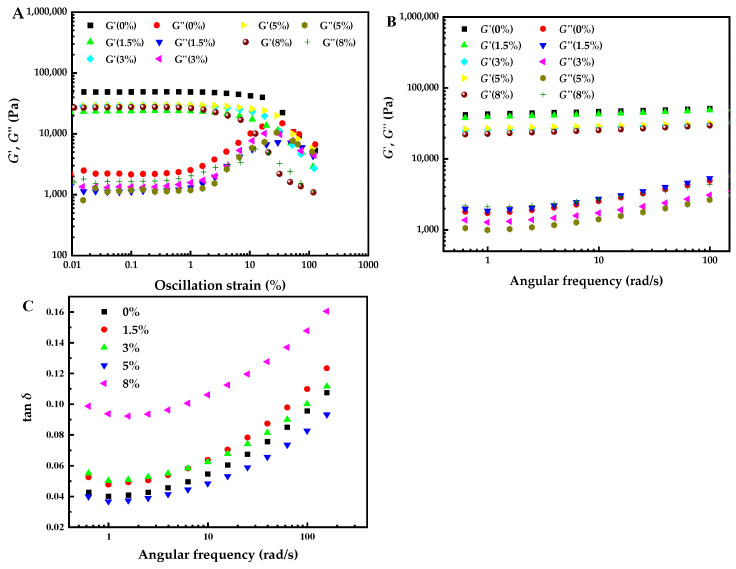
Effects of different NaCl additions on rheological properties of MBSS: (**A**) shear strain curve; (**B**,**C**) small oscillating scanning curve at 1% strain amplitude.

In addition to texture measurement, the cooked MBSS was subjected to rheological measurements which could reflect changes in the gel network structure under stress. From the results of the amplitude experiments, the modulus vs. strain relationship was plotted as linear viscoelastic (LVE) with the following non-linear viscoelastic regions. In the LVE region, the storage modulus (*G*′) and loss modulus (*G*′′) remained constant, which indicated that the strain sweep test was conducted without destroying the structure of the material. As the oscillation amplitude increased, the strain which *G*′ started to decrease sharply was defined as the critical strain, which was associated with the deformability of the material [45]. As shown in Figure 3A, MBSS without NaCl had a broad LVE region and could be considered a strong gel. In contrast, the LVE region of MBSS with NaCl was narrower, indicating that the gel strength and deformability had decreased. This was probably due to the weaker gel network induced by the interaction of salt ions and MBS molecular chains. Furthermore, it was expressed that the strain value at the LVE region for MBSS samples with different NaCl concentrations was within the range of 0.01–1.0%.

The frequency sweep test is usually adopted to distinguish the type of the dispersions, such as dilute solution, concentrated solution, week gel, and strong gel [46,47]. Figure 3B shows changes in the *G*′ and *G*′′ of MBSS with different NaCl concentrations as a function of angular frequency. Within the angular frequency range (0.63–157.08 rad/s), *G*′ was always higher than *G*′′, and no crossover point occurred. This demonstrated that all MBSS samples exhibited more solid-like behavior [40]. *G*′ increased slowly with the increasing frequency, indicating its slight frequency-dependency. However, *G*′′ exhibited very weak frequency-dependency at low frequencies (0.63–1.58 rad/s), but it had higher dependency at high frequencies (1.58–157.08 rad/s). Moreover, the addition of 1.5% (*w*/*w*) NaCl had little effect on the *G*′ of MBSS, but the *G*′ of MBSS generally decreased as the concentration NaCl increases (3–8%, *w*/*w*). The *G*′′ of MBSS containing NaCl (1.5% and 8%, *w*/*w*) was almost close to that without NaCl, while the *G*′′ of MBSS containing NaCl (3% and 5%, *w*/*w*) was lower than that without NaCl. The tan *δ* was defined by *G*′′/*G*′. For all the MBSS samples, tan *δ* values were less than 1 in all of the tested frequencies, indicating that cooked MBSS exhibited primarily elastic gel behavior. As was examined more closely, tan *δ* decreased slightly in the range of 0.63–1.58 rad/s and then continued to increase in the range of 1.58–157.08 rad/s. Meanwhile, NaCl showed marked influence on the tan *δ* value. Compared to MBSS without NaCl, MBSS containing 1.5%, 3%, and 8% (*w*/*w*) NaCl had greater tan *δ*, but MBSS containing 5% (*w*/*w*) NaCl had lower tan *δ*. MBSS containing 5% (*w*/*w*) NaCl had the lowest *G*′ and the greatest elastic behavior among the samples.

### 3.5. Sensory Analysis

**Figure 4:**
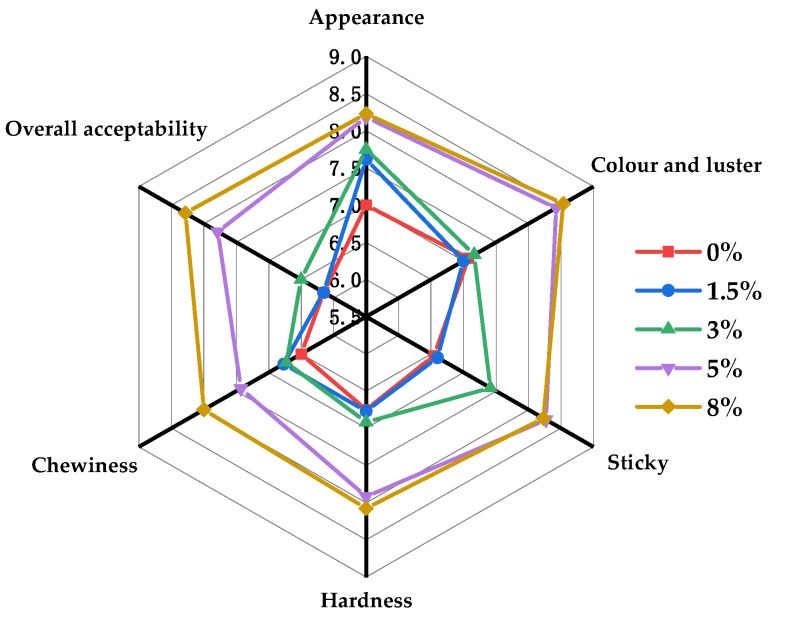
Sensory evaluation of cooked MBSS with different amounts of NaCl.

Six sensory attributes were generated for sensory testing of cooked MBSS. As shown in Figure 4, the sensory characteristics of cooked MBSS were changed by the addition of NaCl. The appearance score was improved with the increasing NaCl content, reflecting a more compact appearance structure. Moreover, the cooked MBSS with 5% and 8% NaCl addition had high scores in color and luster, stickiness, hardness, and chewiness. The results suggested that NaCl promoted the formation of a uniform starch gel network, reduced the surface roughness, and enhanced the texture smoothness of cooked MBSS. Due to the improved appearance and textural attributes, NaCl markedly improved the overall acceptability of the cooked MBSS.

### 3.6. Effect of NaCl on the Short-Range Order of Mung Bean Starch Sheet Jelly

**Figure 5:**
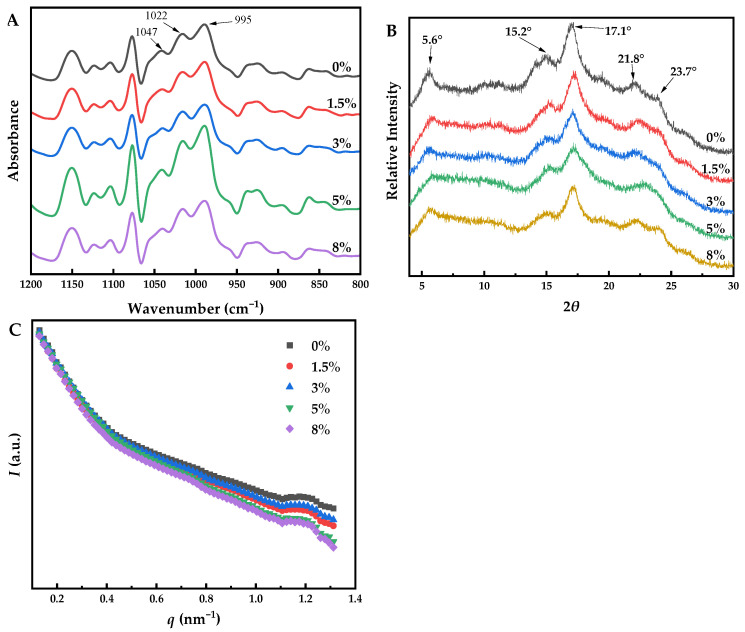
IR spectra (800–1200 cm^−1^) (**A**); X-ray diffraction patterns (**B**); and SAXS patterns (**C**) of dried MBSS with different NaCl addition amounts (0–8%).

ATR-FTIR is a surface analytical technique that can obtain information about the outer regions (~2 μm) of starch materials [48]. In the region of 800–1200 cm^−1^, the FTIR spectra is sensitive to the conformational characteristics of starch. The FT-IR spectral region (800–1200 cm^−1^) of MBSS with or without NaCl after baseline correction and deconvolution is shown in Figure 5A. Particularly, the IR bands at 1047 and 995 cm^−1^ were associated with the ordered structure and crystallinity of starch polymers; whereas the band at 1022 cm^−1^ was reflected of the vibrational modes of starch’s amorphous structure. The ratio of absorbance 1047/1022 cm^−1^ (*R*_1047/1022_) was used to quantify the degree of order in starch, and the ratio of absorbance 1022/995 cm^−1^ (*R*_1022/995_) was used to reflect the ratio of amorphous to ordered structures in the starch granules [49,50]. According to Table 3, the *R*_1047/1022_ and *R*_1022/995_ values were insignificantly varied (*p* > 0.05) among MBSS samples containing 0%, 1.5%, and 3% (*w*/*w*) NaCl. As the addition of NaCl further increased (5% and 8%, *w*/*w*), *R*_1047/1022_ significantly decreased (*p* < 0.05) but *R*_1022/995_ significantly increased (*p* < 0.05). It was indicated that the high level of NaCl suppressed the rearrangement of double helices of starch in MBSS. This can probably be explained by the fact that sodium ions could disrupt the hydrogen bonds among starch molecules, interact with hydroxyl groups, and damage the stability of the double helix structure, leading to the reduction of *R*_1047/1022_ and the degree of order [16]. Moreover, the radically increased *R*_1022/995_ is ascribed to the increase in the mobility of starch molecules and the interaction between starch and water.

### 3.7. Effect of NaCl on the Crystalline Structure of Mung Bean Starch Sheet Jelly

The X-ray diffraction pattern of MBSS with different NaCl additions is shown in Figure 5B. The crystal structure of starch in MBSS without NaCl showed a typical C-type, with strong diffraction peaks at 2*θ* = 5.6°, 15.2°, 17.1°, 21.8°, and 23.7°. The position of the diffraction peaks was unchanged by the addition of NaCl with different concentrations (0–8%, *w*/*w*), indicating that NaCl has no obvious effect on the type of crystallinity. However, the relative crystallinity (RC) of the sample gradually decreased from 38.73% to 31.68% when NaCl concentration increased from 0% to 8% (Table 3). Similar findings have been reported by Tao et al. [51], who reported that Na_2_CO_3_ lowered the crystallinity of wheat starch from 30.11% to 23.13%. Similarly, Yang et al. [16] reported that the XRD crystallinity of potato starch was reduced from 30.13% to 28.61% when the addition amount of NaCl increased from 0% to 10%. Yang et al. [16] suggested that sodium ions entered the semi-crystalline region to dissociate the hydrogen bonds and move the adjacent double helices, which led to a significant decrease in the crystallinity of starch.

### 3.8. Effect of NaCl on the Supramolecular Structure of Dried Mung Bean Starch Sheet Jelly

SAXS is a powerful analytical technique used to determine the nanoscale structure of material by using specialized instruments to detected the X-rays scattered from a sample at low angles, typically in the range of 0.1° to 5° [52]. In this study, the SAXS technique was used to investigate the nanoscale structure of MBSS with different NaCl contents. The *I* (*q*)~*q* curve was obtained by Fit2D and Origin software and is depicted in Figure 5C. The position of the scattering peak was thought to be caused by the lamellar structure of the alternating crystalline and amorphous regions and to correspond to Bragg spacing [53]. The scattering peak intensity was related to the difference in electron density between the lamellar crystalline region and the amorphous region [53]. In Figure 5C, it is shown that the scattering peak of the MBSS was in the range of 1.178–1.197 nm^−1^. The scattering peak intensity was decreased by adding NaCl, which indicated that NaCl hindered the orientation of the double helix of the semi-crystalline lamellar region of MBSS. According to the Bragg formula, *d* = 2π/*q*, Bragg spacing, or the thickness of the semi-crystalline layer (*d*), could be obtained. As shown in Table 3, *d* gradually increased from 5.33 nm to 5.42 nm with the increase in NaCl concentration. The increase in *d* in the case of MBSS was probably due to the reduction in the order [54]. Probably, NaCl promoted the movement of double helices during the drying of MBSS, which could induce the disorganization of starch semi-crystalline lamellae [24,55].

Fractal geometry is a mathematical concept that is used for the description of the structural heterogeneity of an object with self-similarity and no feature length [56]. The parameter, fractal dimension (*D*), is usually described as the quantitative characterization of its irregularity [52]. According to the fractal analysis technique applied, irregular objects can be characterized by the surface fractal dimension (*D*_s_) and the mass fractal dimension (*D*_m_). In theory, the SAXS intensity from a fractal object has a simple power law equation [52]:

*I* (*q*)*~q*^−α^, (4)

where *I* is the intensity of scattering and *q* is the scattering vector. The exponent *α* is the characteristic parameter related to the fractal dimension, which can be used to calculate *D* values.

The values of *α* and *D* can be calculated via the following formula:

ln *I* (*q*) = ln *I*_0_ − αln *q*. (5)

When −4 < *α* < −3, the scattering can be classified as a reflection from the surface, which is regarded as a “surface fractal”. This is used to indicate the degree of smoothness of scattering objects [51]. The surface fractal dimension (*D*_s_) can be calculated via the following formula:

*D*_s_ = 6 + *α*. (6)

When −3 < α < −1, the scattering can be judged to be a “mass fractal”, which is classified as an indicator of compactness [50]. The mass fractal dimension (*D*_m_) can be calculated via the following formula:

*D*_m_ = −*α*. (7)

According to the relationship between *I* (*q*)*~q*^−^^α^, the data were fitted to obtain the *α* value and fractal dimension of each sample. As shown in Table 3, it was found that the *α* of the starch of MBSS with 0–3% (*w*/*w*) NaCl content ranged from −1 to −3, so the starch could be classified as a “mass fractal”. Moreover, the *D*_m_ of the starch of MBSS ranged from 2.71 to 2.84, and it increased with the increase in NaCl content, suggesting that the starch structure in NaCl-containing MBSS was more compact than the sample without NaCl. As the NaCl concentration increased from 5% to 8% (*w*/*w*), the *D*_s_ increased from 2.95 to 2.99, suggesting the presence of a “surface fractal” structure in the samples [57]. This meant that the semi-crystalline lamellae structure of MBSS with greater NaCl content was more compact and complete.

### 3.9. Correlations between Structure and Properties of Mung Bean Starch and Mung Bean Starch Sheet Jelly

In order to acquire a deeper comprehension of the relationships between the structure and properties of starch in MBSS, Pearson correlation analysis was performed, and the results are shown in Table 4. Notably, *R*_1047/1022_ and RC were negatively correlated with the gelatinization temperature (*T*_o_, *T*_p_, *T*_c_, and Δ*T*), but they were positively correlated with Δ*H* (*p* < 0.05). However, the correlation of *R*_1022/995_ and *d* with the thermal parameters showed the opposite trend to *R*_1047/1022_ and RC. The results suggested that the molecular structure order (short-range order degree and long-range order degree) and lamellar structure order were mainly responsible for the thermal energy consumption during MBS gelatinization, which is in agreement with the previous studies [53,58,59]. *R*_1047/1022_ and RC was negatively correlated with the pasting properties (PV, TV, BD, FV, TTPV, and PT), while *R*_1022/995_ and *d* were positively correlated with them. Furthermore, *R*_1047/1022_ and RC were negatively correlated with the cooking properties (cooking yield and cooking loss), while *R*_1022/995_ and *d* were positively correlated with them. *R*_1047/1022_ and RC were positively correlated with the hardness of cooked MBSS, while they were negatively correlated with springiness of cooked MBSS. However, the correlation of *R*_1022/995_ and *d* with the texture properties (hardness and springiness) showed the opposite trend. Similar results were observed in studies of cassava and yam starch gels, namely, that the order structures showed a correlation with texture properties [60]. Moreover, Dereje et al. [61] found that high short-range order in starch would limit the starch gelatinization and promote successive starch retrogradation, resulting in a difficult-to-gelatinize starch gel with high hardness. *R*_1022/995_ and *d* were positively correlated with the toughness of dried MBSS, while *R*_1047/1022_ and RC were negatively correlated with it, indicating that the order range structures of starch in MBSS might enhance the brittleness of dried MBSS, reducing its deformation resistance. For the sensory properties, overall acceptability was found to be negatively correlated with *R*_1047/1022_ (r = −0.95; *p* < 0.05) and RC (r = −0.95; *p* < 0.05), but positively correlated with *R*_1022/995_ (r = 0.94; *p* < 0.05) and *d* (r = 1.00; *p* < 0.01). It was suggested that the order range structures of starch play an important role in the sensory quality of MBSS.

## 4. Conclusions

In summary, NaCl markedly affected the physicochemical properties of MBS and the quality of MBSS. First, NaCl had an impact on the making and drying process of MBSS. The addition of NaCl increased the gelatinization temperature (*T*_o_, *T*_p_, *T*_c_, and Δ*T*) and pasting parameters of MBS but decreased the Δ*H* of MBS. Moreover, NaCl apparently affected the drying of MBSS. Regarding the cooking properties, the NaCl addition improved the cooking yield and cooking loss of MBSS. In terms of the texture properties, NaCl decreased the hardness of cooked MBSS, but it had no significant effect (*p* > 0.05) on the springiness of cooked MBSS. Rheological measurements of the cooked MBSS suggested that the LVE region decreased with the addition of NaCl. Moreover, *G*″ and tan *δ* were more frequency-dependent than *G*′ in the range of 0.63–157.08 rad/s. Furthermore, the toughness of dried MBSS was enhanced by NaCl, which made it less prone to fragmentation. The addition of NaCl up to 8% (*w*/*w*) progressively increased the overall acceptability of cooked MBSS. These results were attributed to the alteration of the starch structure in MBSS as affected by NaCl addition. NaCl decreased the order structure of starch in MBSS, as proved by the results of ATR-FTIR, XRD, and SAXS. Correlation analysis showed that cooking yield, springiness, and overall acceptability exhibited a significant positive correlation with the thickness of the semi-crystalline layer (*d*), but they showed a significant negative correlation with relative crystallinity (RC) and the degree of order (*R*_1047/1022_). This study could provide a reference for the application of NaCl in the processing of starch gel-based products with improved mechanical, sensory, and nutritional attributes. Moreover, the present study provides a recommendation for the use of NaCl in the development of starch-based edible films and coatings.

## Figures and Tables

**Table 1:** Thermal and pasting properties of MBS with different NaCl additions ^a^.

Parameters	NaCl Addition Amount (%)				
	0	1.5	3	5	8
Thermal properties ^b^					
*T*_o_ (°C)	55.79 ± 0.07 d	57.02 ±0.03 c	57.50 ± 0.16 b	57.92 ± 0.16 a	58.17 ± 0.07 a
*T*_p_ (°C)	66.03 ± 0.11 d	69.45 ±0.01 c	69.41 ± 1.00 c	72.01 ± 0.33 b	73.56 ± 0.12 a
*T*_c_ (°C)	74.97 ± 0.73 c	79.52 ±0.53 b	76.32 ± 1.52 bc	85.67 ± 1.39 a	89.23 ± 3.49 a
Δ*T* (°C)	19.19 ± 0.80 b	22.49 ±0.50 b	18.82 ± 1.49 b	27.75 ± 1.55 a	31.06 ± 3.56 a
Δ*H* (J/g)	9.63 ± 0.37 a	9.41 ± 0.61 a	9.58 ± 0.75 a	8.84 ± 0.45 ab	7.86 ± 0.34 b
Pasting properties ^c^					
PV (cP)	3162.5 ± 26.16 d	3320.0 ± 5.66 c	3364.0 ± 21.21 bc	3476.5 ± 126.57 b	4428.7 ± 50.40 a
TV (cP)	2478.5 ± 123.74 a	2461.0 ± 14.14 a	2518.5 ± 10.60 a	2572.0 ± 74.95 a	2633.3 ± 99.45 a
BD (cP)	684.0 ± 97.58 c	859.0 ± 26.87 b	845.5 ± 10.61 b	904.5 ± 51.62 b	1795.3 ± 54.86 a
FV (cP)	3962.5 ± 214.25 b	4724.0 ± 28.28 a	4727.5 ± 86.97 a	4960.0 ± 246.07 a	4206.0 ± 40.58 b
SB (cP)	1484.0 ± 338.00 b	2263.0 ± 28.28 a	2209.0 ± 76.37 a	2388.0 ± 171.12 a	1572.7 ± 93.03 b
TTPV (min)	4.97 ± 0.14 b	5.00 ± 0.00 b	5.03 ± 0.05 b	4.93 ± 0.09 b	5.24 ± 0.10 a
PT (°C)	74.75 ± 0.08 b	75.5 ± 0.42 ab	76.75 ± 0.49 a	76.70 ± 0.64 a	75.33 ± 0.65 b

^a^ Mean values signed with different letters in particular rows are significantly different at a 0.05 level of confidence. ^b^ *T*_o_, *T*_p_, and *T*_c_ represent onset, peak, and completion gelatinization temperatures, respectively. Δ*T* means the difference between *T*_o_ and *T*_c_; Δ*H* denotes gelatinization enthalpies. ^c^ PV, TV, BD, FV, SB, TTPV, PT represent peak viscosity, trough viscosity, breakdown, final viscosity, setback, time to peak viscosity, and pasting temperature, respectively.

**Table 2:** Cooking properties and textural properties of MBSS with different NaCl addition amounts.

Parameters	NaCl Addition Amount (%)				
	0	1.5	3	5	8
Cooking properties					
Cooking yield (%)	177.81 ± 5.42 d	191.73 ± 13.29 cd	202.05 ± 5.17 c	277.59 ± 18.04 b	317.69 ± 11.79 a
Cooking loss (%)	0.23 ± 0.13 c	1.95 ± 0.91 b	1.92 ± 0.84 b	2.60 ± 0.73 a	5.91 ± 0.96 a
Textural properties					
Hardness (N)	516.23 ± 31.36 a	505.90 ± 43.92 ab	459.39 ± 55.45 abc	444.75 ± 73.29 bc	387.70 ± 48.23 c
Springiness	0.823 ± 0.091 a	0.849 ± 0.143 a	0.859 ± 0.073 a	0.887 ± 0.072 a	0.898 ± 0.108 a

Mean values signed with different lowercase letters in particular rows are significantly different at a 0.05 level of confidence.

**Table 3:** The ordered structure parameters of MBSS with different NaCl addition amounts.

Parameters	NaCl Addition Amount (%)				
	0	1.5	3	5	8
IR absorbance ratio					
*R*_1047/1022_	1.03 ± 0.07 a	1.00 ± 0.05 ab	0.94 ± 0.03 ab	0.89 ± 0.01 bc	0.78 ± 0.07 c
*R*_1022/995_	0.39 ± 0.03 c	0.40 ± 0.01 bc	0.45 ± 0.04 abc	0.47 ± 0.01 ab	0.51 ± 0.03 a
RC (%)	35.57 ± 0.57 a	33.78 ± 0.37 b	32.25 ± 0.73 c	30.41 ± 0.80 d	27.86 ± 0.09 e
SAXS parameter					
*d* (nm)	5.33	5.33	5.34	5.39	5.42
*D*_m_	2.71	2.80	2.84	-	-
*D*_s_	-	-	-	2.95	2.99

*R*_1047/1022_ means the ratio of absorbance 1047/1022 cm^−1^; *R*_1022/995_ means the ratio of absorbance 1047/1022 cm^−1^; RC means the relative crystallinity; *d* means the Bragg spacing; *D*_m_ means the mass fractal dimension; and *D*_s_ means the surface fractal dimension. Mean values signed with different letters in particular rows are significantly different at a 0.05 level of confidence.

**Table 4:** The correlation coefficients of Pearson correlation between structural and functional properties of MBSS.

	*R*_1047/1022_	*R*_1022/995_	RC	*d*
*T*_o_	−0.87	0.90 *	−0.92 *	0.79
*T*_p_	−0.92 *	0.91 *	−0.97 **	0.89 *
*T*_c_	−0.90 *	0.84	−0.91 *	0.95 *
Δ*T*	−0.87	0.80	−0.88	0.94 *
Δ*H*	0.94 *	−0.87	0.91 *	−0.96 **
PV	−0.92 *	0.86	−0.89 *	0.88 *
TV	−0.98 **	0.97 **	−0.95 *	0.98 **
BD	−0.90 *	0.82	−0.86	0.85
FV	−0.06	0.16	−0.20	0.01
SB	0.10	−0.01	−0.34	−0.15
TTPV	−0.74	0.66	−0.68	0.62
PT	−0.23	0.39	−0.33	0.14
Cooking yield	−0.96 **	0.94 *	−0.96 **	1.00 **
Cooking loss	−0.95 *	0.89 *	−0.94 *	0.89 *
HD	0.99 **	−0.99 **	0.98 **	−0.93 *
SP	−0.94 *	0.95 *	−0.98 **	0.92 *
TH	−0.87	0.90 *	−0.92 *	0.79
AP	−0.88 *	0.90 *	−0.94 *	0.84
CL	−0.89 *	0.88 *	−0.89 *	0.98 **
ST	−0.91 *	0.95 *	−0.93 *	0.91 *
HN	−0.92 *	0.91 *	−0.92 *	0.98 **
CH	−0.96 **	0.92 *	−0.96 **	0.98 **
OA	−0.95 *	0.94 *	−0.95 *	1.00 **

HD and SP mean hardness and springiness of texture properties; TH means toughness of sensory quality in dried MBSS; AP, CL, ST, HN, CH, and OA mean appearance, color and luster, sticky, hardness, chewiness, and overall acceptability, respectively. * Correlations are significant at *p* < 0.05. ** Correlations are significant at *p* < 0.01.

## Data Availability

Data will be made available on request.

## Appendix A

**Table A1:** Sensory scoring items and scoring criteria for MBSS.

Evaluation Index	Specific Feature Description
Toughness ^a^	Excellent toughness, hard to break (8~10)
	Immediate toughness, hard to break (5~7)
	Poor toughness, easy to break (0~4)
Appearance ^b^	Dense and smooth surface structure (8~10)
	Slightly loose structure (5~7)
	Loose and rough structure (0~4)
Color and luster ^b^	Transparent and rich luster (8~10)
	Immediate transparency, immediate luster (5~7)
	Poor transparency, poor luster (0~4)
Stickiness ^b^	Slipperiness (8~10)
	Almost not sticky (5~7)
	Sticky (0~4)
Hardness ^b^	Appropriate strength (8~10)
	Slightly soft or hard (5~7)
	Too soft or too hard (0~4)
Chewiness ^b^	Chewy (8~10)
	Slightly chewy (5~7)
	Poor chewiness (0~4)
Overall acceptability ^b^	Preferably (8~10)
	Normal (5~7)
	Poor (0~4)

^a^ Means that the evaluation index was for the MBSS after drying. ^b^ Means that the evaluation indexes were for the MBSS after cooking.

## References

[1] Tan H.L., Tan T.C., Easa A.M. (2018). Comparative study of cooking quality, microstructure, and textural and sensory properties between fresh wheat noodles prepared using sodium chloride and salt substitutes. LWT.

[2] Hutton T. (2002). Sodium technological functions of salt in the manufacturing of food and drink products. Br. Food J..

[3] You B., Yang S., Yu J., Xian W., Deng Y., Huang W., Li W., Yang R. (2021). Effect of thermal and dry salt-curing processing on free and bound phenolics and antioxidant activity in *Prunus mume* fruits together with the phenolic bioaccessibility. LWT.

[4] Zhang J., Zhang W., Ma C., Cai J. (2023). Evaluation of ultrasound-assisted process as an approach for improving the overall quality of unsmoked bacon. Ultrason. Sonochem..

[5] Petracci M., Bianchi M., Mudalal S., Cavani C. (2013). Functional ingredients for poultry meat products. Trends Food Sci. Technol..

[6] Aaslyng M.D., Vestergaard C., Koch A.G. (2014). The effect of salt reduction on sensory quality and microbial growth in hotdog sausages, bacon, ham and salami. Meat Sci..

[7] Jin G., He L., Yu X., Zhang J., Ma M. (2013). Antioxidant enzyme activities are affected by salt content and temperature and influence muscle lipid oxidation during dry-salted bacon processing. Food Chem..

[8] McCann T.H., Day L. (2013). Effect of sodium chloride on gluten network formation, dough microstructure and rheology in relation to breadmaking. J. Cereal Sci..

[9] Li M., Sun Q.J., Han C.W., Chen H.H., Tang W.T. (2018). Comparative study of the quality characteristics of fresh noodles with regular salt and alkali and the underlying mechanisms. Food Chem..

[10] Moraru C.I., Kokini J.L. (2003). Nucleation and expansion during extrusion and microwave heating of cereal foods. Compr. Rev. Food Sci. Food Saf..

[11] Norton A.D., Greenwood R.W., Noble I., Cox P.W. (2011). Hot air expansion of potato starch pellets with different water contents and salt concentrations. J. Food Eng..

[12] Tan H.Z., Li Z.G., Tan B. (2009). Starch noodles: History, classification, materials, processing, structure, nutrition, quality evaluating and improving. Food Res. Int..

[13] Li W., Bai Y., Zhang Q., Hu X., Shen Q. (2011). Effects of potassium alum addition on physicochemical, pasting, thermal and gel texture properties of potato starch. Int. J. Food Sci. Technol..

[14] Li Z., Zhang Y., Ai Z., Fan H., Wang N., Suo B. (2019). Effect of potassium alum addition on the quality of potato starch noodles. J. Food Sci. Technol..

[15] Rengel Z. (2004). Aluminium cycling in the soil-plant-animal-human continuum. Biometals.

[16] Yang S., Dhital S., Zhang M.N., Wang J., Chen Z.G. (2022). Structural, gelatinization, and rheological properties of heat-moisture treated potato starch with added salt and its application in potato starch noodles. Food Hydrocoll..

[17] Zhuang Y., Wang Y., Yang H. (2024). Effects of cation valence on swelling power, solubility, pasting, gel strength characteristics of potato starch. Food Chem..

[18] Zheng L., Ren A., Liu R., Xing Y., Yu X., Jiang H. (2022). Effect of sodium chloride solution on quality of 3D-printed samples molded using wheat starch gel. Food Hydrocoll..

[19] Saha S., Roy A. (2020). Puffed rice: A materialistic understanding of rice puffing and its associated changes in physicochemical and nutritional characteristics. J. Food Process Eng..

[20] Huang Y., Bao X., Li P., Zhan L., Wu H., Chen P. (2022). Effect of NaCl addition on alcohol-alkali-treated waxy rice starch: Structural and physicochemical functionality. Food Chem..

[21] Kaur K., Kaur G., Singh A. (2023). Water chestnut starch: Extraction, chemical composition, properties, modifications, and application concerns. Sustain. Food Technol..

[22] Yan S., Liu C., Li S., Shen Q. (2010). The role of NaCl or KCl act on precipitation rate and some properties of mung bean starch. Int. J. Food Sci..

[23] Chen C., Li G., Corke H., Zhu F. (2023). Physicochemical properties of starch in sodium chloride solutions and sucrose solutions: Importance of starch structure. Food Chem..

[24] Yuan T., Ye F., Chen T., Li M., Zhao G. (2022). Structural characteristics and physicochemical properties of starches from winter squash (*Cucurbita maxima* Duch.) and pumpkin (*Cucurbita moschata* Duch. ex Poir.). Food Hydrocoll..

[25] He Y., Ye F., Zhang Z., Zou Y., Li S., Chen J., Zhao G. (2023). Unraveling the regulating mechanisms of moisture content in the puffing of sweet potato starch gel. Int. J. Biol. Macromol..

[26] Chen J.Y., Zhang H., Miao Y. (2014). The effect of quantity of salt on the drying characteristics of fresh noodles. J. Agric. Sci..

[27] Yeoh S.Y., Lubowa M., Tan T.C., Murad M., Easa A.M. (2020). The use of salt-coating to improve textural, mechanical, cooking and sensory properties of air-dried yellow alkaline noodles. Food Chem..

[28] Pu H., Wei J., Wang L.E., Huang J., Chen X., Luo C., Liu S., Zhang H. (2017). Effects of potato/wheat flours ratio on mixing properties of dough and quality of noodles. J. Cereal Sci..

[29] Rong L., Shen M., Wen H., Xiao W., Li J., Xie J. (2022). Effects of xanthan, guar and *Mesona chinensis* Benth gums on the pasting, rheological, texture properties and microstructure of pea starch gels. Food Hydrocoll..

[30] Xu H.Y., Chen X.W., Li J., Bi Y.L. (2023). Approach to evaluate the sensory quality deterioration of chicken seasoning using characteristic oxidation indicators. Food Chem. X.

[31] Guo X., Gu F., Li Y., Zhang Q., Hu R., Jiao B., Wang F., Wang Q. (2023). Precooking treatments affect the sensory and tensile properties of autoclaved recooked noodles via moisture distribution and protein structure. Food Chem..

[32] Li S., Ye F., Zhou Y., Lei L., Zhao G. (2019). Rheological and textural insights into the blending of sweet potato and cassava starches: In hot and cooled pastes as well as in fresh and dried gels. Food Hydrocoll..

[33] Qiao D., Tu W., Liao A., Li N., Zhang B., Jiang F., Zhong L., Zhao S., Zhang L., Lin Q. (2019). Multi-scale structure and pasting/digestion features of yam bean tuber starches. Carbohydr. Polym..

[34] Li E., Lv J., Huo D., Jia B., Li C. (2023). Importance of amylose chain-length distribution in determining starch gelatinization and retrogradation property of wheat flour in the presence of different salts. Carbohydr. Polym..

[35] Day L., Fayet C., Homer S. (2013). Effect of NaCl on the thermal behaviour of wheat starch in excess and limited water. Carbohydr. Polym..

[36] Hong Y., Zhu L., Gu Z. (2014). Effects of sugar, salt and acid on tapioca starch and tapioca starch-xanthan gum combinations. Starch-Stärke.

[37] Zhang T., Li K., Ding X., Sui Z., Yang Q.Q., Shah N.P., Liu G., Corke H. (2021). Starch properties of high and low amylose proso millet (*Panicum miliaceum* L.) genotypes are differentially affected by varying salt and pH. Food Chem..

[38] Zhou H., Wang C., Shi L., Chang T., Yang H., Cui M. (2014). Effects of salts on physicochemical, microstructural and thermal properties of potato starch. Food Chem..

[39] Zhiguang C., Qi Y., ZhaoGuo T., Rui Z., Junrong H., Huayin P., Haixia Z. (2023). The effect rules of MgCl2 and NaCl on the properties of potato starch: The inflection point phenomenon. Int. J. Biol. Macromol..

[40] Luo Y., Shen M., Han X., Wen H., Xie J. (2020). Gelation characteristics of *Mesona chinensis* polysaccharide-maize starches gels: Influences of KCl and NaCl. J. Cereal Sci..

[41] Sudhakar V., Singhal R.S., Kulkarni P.R. (1996). Effect of salts on interactions of starch with guar gum. Food Hydrocoll..

[42] Shukla B.D., Singh S.P. (2007). Osmo-convective drying of cauliflower, mushroom and greenpea. J. Food Eng..

[43] Farahnaky A., Askari H., Majzoobi M., Mesbahi G.H. (2010). The impact of concentration, temperature and pH on dynamic rheology of psyllium gels. J. Food Eng..

[44] Ciaramitaro V., Piacenza E., Meo P.L., Librici C., Calvino M.M., Conte P., Lazzara G., Martino D.F.C. (2023). From micro to macro: Physical-chemical characterization of wheat starch-based films modified with PEG200, sodium citrate, or citric acid. Int. J. Biol. Macromol..

[45] Pourfarzad A., Yousefi A., Ako K. (2021). Steady/dynamic rheological characterization and FTIR study on wheat starch-sage seed gum blends. Food Hydrocoll..

[46] Yousefi A.R., Razavi S.M. (2015). Dynamic rheological properties of wheat starch gels as affected by chemical modification and concentration. Starch-Stärke.

[47] Chen J., Shi D., Yang Z., Dong K., Kaneko D., Chen M. (2021). Hand-extended noodle inspired physical conjoined-network organohydrogels with anti-freezing, high stiffness and toughness properties. J. Mater. Sci. Technol..

[48] Sevenou O., Hill S.E., Farhat I.A., Mitchell J.R. (2002). Organisation of the external region of the starch granule as determined by infrared spectroscopy. Int. J. Biol. Macromol..

[49] Van Soest J.J., Tournois H., de Wit D., Vliegenthart J.F. (1995). Short-range structure in (partially) crystalline potato starch determined with attenuated total reflectance Fourier-transform IR spectroscopy. Carbohydr. Res..

[50] Zhou D., Ma Z., Yin X., Hu X., Boye J.I. (2019). Structural characteristics and physicochemical properties of field pea starch modified by physical, enzymatic, and acid treatments. Food Hydrocoll..

[51] Tao H., Li M., Deng H.D., Ren K.X., Zhuang G.Q., Xu X.M., Wang H.L. (2019). The impact of sodium carbonate on physico-chemical properties and cooking qualities of starches isolated from alkaline yellow noodles. Int. J. Biol. Macromol..

[52] Li Z.H. (2013). A program for SAXS data processing and analysis. Chinese Phys. C.

[53] Lin L., Guo D., Zhao L., Zhang X., Wang J., Zhang F., Wei C. (2016). Comparative structure of starches from high-amylose maize inbred lines and their hybrids. Food Hydrocoll..

[54] Shamai K., Shimoni E., Bianco-Peled H. (2004). Small angle X-ray scattering of resistant starch type III. Biomacromolecules.

[55] Liu C., Jiang Y., Liu J., Li K., Li J. (2021). Insights into the multiscale structure and pasting properties of ball-milled waxy maize and waxy rice starches. Int. J. Biol. Macromol..

[56] Suzuki T., Chiba A., Yarno T. (1997). Interpretation of small angle X-ray scattering from starch on the basis of fractals. Carbohydr. Polym..

[57] Ma Z., Ma M., Zhou D., Li X., Hu X. (2019). The retrogradation characteristics of pullulanase debranched field pea starch: Effects of storage time and temperature. Int. J. Biol. Macromol..

[58] Tian Y., Liu X., Kirkensgaard J.J.K., Khakimov B., Enemark-Rasmussen K., Hebelstrup K.H., Blennow A., Zhong Y. (2024). Characterization of different high amylose starch granules. Part I: Multi-scale structures and relationships to thermal properties. Food Hydrocoll..

[59] Tian Y., Qu J., Zhou Q., Ding L., Cui Y., Blennow A., Zhong Y., Liu X. (2022). High pressure/temperature pasting and gelling of starch related to multilevel structure-analyzed with RVA 4800. Carbohydr. Polym..

[60] Zou J., Li Y., Wang F., Su X., Li Q. (2023). Relationship between structure and functional properties of starch from different cassava (Manihot esculenta Crantz) and yam (*Dioscorea opposita* Thunb) cultivars used for food and industrial processing. LWT.

[61] Dereje B. (2021). Composition, morphology and physicochemical properties of starches derived from indigenous Ethiopian tuber crops: A review. Int. J. Biol. Macromol..

